# Prevalence and Distribution of Oral Mucosal Lesions and Normal Variants among Nepalese Population

**DOI:** 10.1155/2023/9375084

**Published:** 2023-10-18

**Authors:** Abhishek Gupta, Parikshya Shrestha, Sijan Poudyal, Sandeep Kumar, Ram Sudan Lamichhane, Surendra Kumar Acharya, Peeyush Shivhare

**Affiliations:** ^1^Department of Oral Medicine and Radiology, Chitwan Medical College, Bharatpur, Chitwan 44207, Nepal; ^2^Department of Oral and Maxillofacial Pathology, KIST Medical College and Teaching Hospital, Lalitpur 44705, Nepal; ^3^Department of Community Dentistry, People's Dental College and Hospital Pvt. Ltd., Kathmandu 44600, Nepal; ^4^Department of Public Health Dentistry, Dental Institute, Rajendra Institute of Medical Sciences, Ranchi, Jharkhand 834009, India; ^5^Department of Oral and Maxillofacial Surgery, KIST Medical College and Teaching Hospital, Lalitpur 44705, Nepal; ^6^Department of Dentistry, All India Institute of Medical Sciences, Patna 801507, India

## Abstract

**Background:**

Oral mucosa is encountered by various lesions and normal variants. Some are not to be worried about, whereas others may be of significance. Knowing the prevalence of oral mucosal lesions in a particular region helps better evaluate, diagnose, and, thus, manage these lesions.

**Objectives:**

To assess the prevalence and distribution of oral mucosal lesions and normal variants among various age groups, genders, and sites of the orofacial region.

**Methods:**

This cross-sectional study was conducted in the Department of Oral Medicine and Radiology, KIST Medical College and Teaching Hospital from January 2021 to March 2021. Three different proformas were designed according to age, gender, and location of lesions for entry as per the WHO's guide. The obtained data were entered into a Microsoft Excel sheet for frequency analysis by SPSS, and the results were tabulated.

**Results:**

Among the records of 16572 (9703 (58.55%) males and 6869 (41.44%) females) OPD patients, 3495 (21.08%) (1934 (55.33%) males and 1561 (44.66%) females) had OMLs and 2314 (13.96%) (1626 (70.26%) males and 688 (29.73%) females) had normal mucosal variants. The most commonly seen OML categories were tobacco-associated lesions, i.e., 2056 (34.07%), tongue lesions, i.e., 1598 (26.48%), oral potentially malignant disorders, i.e., 815 (13.50%), ulcers i.e., 728 (12.06%), and infectious lesions, i.e., 256 (4.24%).

**Conclusion:**

The Nepalese population has a wide range of oral mucosal lesions and normal variants, and this study has attempted to have baseline data for the same. The most common OML was smoker's melanosis.

## 1. Introduction

The orofacial region is affected by various oral mucosal lesions (OMLs). Oral mucosa is explicitly affected by seven oral potentially malignant disorders [1]. Various other OMLs include several types of cysts, benign and malignant tumours, inflammatory lesions, lesions associated with tobacco, areca nut [2], betel nut chewing [3], and others; immune-mediated lesions like recurrent aphthous stomatitis (RAS) and oral pemphigus, which pose a challenge in the management and may become life-threatening if early diagnosis fails. These lesions cause disturbance in day-to-day activities as they interfere with the consumption of food, causing pain, burning sensation, facial asymmetry, and others. In contrast, other normal variants of oral mucosa do not cause harm but can be misdiagnosed as a potentially life-threatening condition. This makes it necessary for us to have the proper knowledge about oral lesions (OLs) and the normal variants for proper management. These lesions vary depending on geography, race, culture, ethnicity, food, or deleterious habits [4]. Therefore, adequate knowledge about the prevalence of oral lesions is of great significance as it helps in the prevention, correct diagnosis, and management.

The prevalence of OMLs and normal variants is between 10.8% and 61.6% among the various populations [5]. Epidemiological studies of OLs are still lacking when compared to the studies of dental caries or periodontal diseases [6], and just two studies based on this topic have been reported in Nepal to date [7, 8]. Those studies were not focused on the prevalence of normal variants and OMLs; instead, they were histological studies done on pathological samples, so they could not find the overall prevalence. This prompted us to conduct a study on the prevalence of oral lesions and normal variants of oral mucosa from previous dental records of patients who had visited the dental hospital in Lalitpur within the provided time frame.

The objectives of this study were to study the prevalence and distribution of various oral mucosal lesions and normal variants among various age groups, genders, and sites of the orofacial region.

## 2. Material and Methods

### 2.1. Study Design

This cross-sectional study was carried out in the Department of Oral Medicine and Radiology, KIST Medical College and Teaching Hospital, from January 2021 to March 2021. Ethical approval was given by the institutional review committee of the same institution. (IRC Ref. No. 077/078/26) The census sampling method was used for all the cases of oral lesions recorded from January 2016 to December 2019, meeting the inclusion criteria.

### 2.2. Inclusion and Exclusion Criteria

Clinically or histologically diagnosed as any of the oral mucosal lesions, along with data of the location/site of the lesion.

Data including clinical/histological diagnosis, age, gender, and site/sites of the lesion were included. Record with incomplete demographic or clinical data was excluded. Also, the same patient was not included again as a new patient, which was done with the help of the unique OPD number provided to each patient.

### 2.3. Data Sources

Data collection was done manually by going through the previous outpatient records of the oral medicine and radiology department. Three different proformas were designed for entry according to age, gender, and location of lesions, which was done taking into consideration the WHO's guide to epidemiology and diagnosis of oral mucosal diseases and conditions [9].

### 2.4. Statistical Methods

The obtained data was entered in a Microsoft Excel sheet for frequency analysis by IBM SPSS Statistics for Windows, version 27 (IBM Corp., Armonk, N.Y., USA), and the results were tabulated.

## 3. Result

Among the records of 16572 OPD patients, 3495 (21.08%) had OMLs, 2314 (13.96%) had normal mucosal variants, and 10763 (64.94%) had normal oral mucosa. A total of 55 types of OMLs were noted among the 10437 lesions. These 50 types of OMLs were categorized into 11 different groups based on common characteristics, and one group was categorized as others, which could not be categorized otherwise. More than 45% of individuals had more than one OMLs and/or more than one normal variant; therefore, the total OMLs were 6033, and the total normal variant was 4404.

Among the 12 categories, the most commonly seen OML categories in descending order were tobacco-associated lesions, i.e., 2056 (34.07%), tongue lesions, i.e., 1598 (26.48%), oral potentially malignant disorders, i.e., 815 (13.50%), ulcers, i.e., 728 (12.06%), infectious, i.e., 256 (4.24%), others, i.e., 172 (2.85%), soft tissue lesions, i.e., 167 (2.76%), typical gingival lesions i.e., 108 (1.79%), bony lesions, i.e., 68 (1.12%), salivary gland lesions, i.e., 39 (0.64%), mucocutaneous lesions, i.e., 17 (0.28%), and malignancy, i.e., 9 (0.14%) (Tables 1(a) and 1(b)).

### 3.1. OMLs and Normal Variants: Gender Distribution

3808 (63.11%) OMLs and 2470 (56.08%) normal variants were found among males. 2225 (36.88%) OMLs and 1934 (43.91%) normal variants were found among females.

Oral potentially malignant disorders 585 (71.77%), malignancy 7 (77.77%), tobacco-associated lesions 1679 (81.66%), and infectious lesion 198 (77.34%) were significantly higher in males as compared to females.

Typical gingival lesions 70 (64.81%) and mucocutaneous 13 (76.47%) were significantly higher among females than males.

The most common OMLs among females were tongue lesions 801 (36%), ulcers 394 (17.70%), tobacco-related 377 (16.94%), OPMDs 230 (10.33%), soft tissue 122 (5.48%), and myositis 97 (4.35%).

The most common OMLs among males were tobacco-related 1679 (44.09%), tongue lesions 797 (20.92%), OPMDs 585 (15.36%), ulcer 334 (8.77%), and infectious 198 (5.19%).

Normal variants were seen more among the males, i.e., 2470 (56.08%), as compared to the females, i.e., 1934 (43.91%) and frictional keratosis 745 (38.52%) were most common among females and linea alba (34.77%) was most common among males (Tables 2(a) and 2(b)).

### 3.2. OMLs and Normal Variants: Age-Wise Distribution

Most of the OMLs were seen in the age group of 41-50 years, i.e., 2670 (25.58%), followed by 51-60 years, i.e., 2373 (22.73%), 31-40 years, i.e., 2358 (22.59%), 21-30 years, i.e., 1508 (14.44%), >60 years, i.e., 1094 (10.48%), and least among 11-20 years i.e., 354 (3.39%), and 0-10 years, i.e., 58 (0.55%).

The most commonly seen OML categories among the age groups of 0-10 years (22, 37.93%), 11-20 years (85, 31.59%), and 21-30years (31.74%) were ulcers; 31-40 years (513, 37.36%), 41-50 years (573, 37.11%), 51-60 years (42.30%), and >60 years (245, 37.00%) were tobacco-associated lesions.

Among the 0-20 years were 629 individuals; 235 (37.36%) individuals had 327 OMLs. The most common OMLs among 0-10 years (21, 36.20%), 11-20 years (83, 30.85%), and 21-30 years (247, 30.15%) were recurrent apthous stomatitis; 31-40 years (213, 15.51%), 41-50 years (232, 15.02%), 51-60 years (221, 17.18%) were smoker melanosis; >60 years (126, 19.03%) was tobacco pouch keratosis.

The most common normal variants among 11-20 years were Fordyce's granules (27, 31.76%); 21-30 years (265, 38.46%), 31-40 years (363, 36.85%), 51-60 years (413, 36.67%), and >60years (233, 53.68%) were frictional keratosis; 41-50 years (419, 37.21%) was linea alba (Tables 3(a) and 3(b)).

### 3.3. OMLs and Normal Variants: Location-Wise Distribution

Most of the lesions involved more than one site. The most common sites for OMLs in descending order were buccal mucosa (2265, 22.23%), tongue (1899, 18.64%), labial mucosa (1704, 16.73%), posterior gingiva (1072, 10.52%), anterior gingiva (1047, 10.27%), vestibule (1014, 9.95%), palate (883, 8.66%), extraoral (211, 2.07%), floor of the mouth (136, 1.33%), mandible (35, 0.34%), and maxilla (2,0.01%).

OPMDs (445, 30.43%), tobacco-associated lesions (1304, 24.18%), ulcers (453, 53.10%), mucocutaneous (6, 22.22%), and soft tissue lesions (50, 27.77%) were seen most commonly on buccal mucosa.

The most common site for normal variants was buccal mucosa (3958, 80.10%), followed by labial mucosa (452, 9.14%), tongue (215, 4.35%), vestibule (113, 2.28%), and gingiva (186, 3.76%).

## 4. Discussion

The prevalence of oral and maxillofacial diseases varies depending on the region, country, and data source [4]. An oral lesion is any abnormal alteration in colour, surface aspect, swelling, or loss of integrity of the oral mucosal surface. Although a proportion of OMLs are benign and require no active treatment, some may present with significant pathology. Besides, OMLs can interfere with the daily quality of life in affected patients [6]. Oral lesions are usually mystified by their aetiology, which may be viral, fungal, bacterial, related, or even without definite aetiology. Understanding the prevalence of oral mucosal lesions may facilitate the prevention, appropriate diagnosis, and prompt treatment of the disease [7]. There is a lack of epidemiological studies based on oral mucosal lesions in the Nepali population, which led us to conduct this study in a tertiary centre in the Lalitpur Region, where patients visit for various dental treatments, as well as are referred from various clinical centres in and out from the Lalitpur Region. To the best of our knowledge, this study was the first attempt to get a prevalence of various oral mucosal lesions among the Nepalese population.

Our study found around 55 types of OMLs (50) and normal variants [5].

The study population was 16572, among which 9703 (58.55%) were males and 6869 (41.44%) were females, which was far more than other studies by Amaral et al. (1075, 430 males and 645 females) [4], El Toum et al. (178, 76 females and 102 males) [6], Bajracharya et al. (111) [8], Gambhir et al. (451) [10], Ali et al. (530, 174 males and 134 females) [11], Blanco DC P et al. (515, 191 males and 324 females) [12], Mumcu et al., (765, 375 males and 390 females) [13], Kumar et al. (1048) [14], Parlak et al. (993) [15], Amaral et al. (1075, 429 males and 646 females) [4], Jahanbani (1167, 1070 males and 97 females) [16], Leite et al. (1385, 648 males and 737 females) [17], Carrard et al. (1586,719 males and 867 females) [18], Oivio et al. (1966) [5], Saraswathi et al. (2017, 1286 males and 731 females) [19], Chiang et al. (2050) [20], Al-Mobeeriek et al. (2552) [21], Mehrotra et al. (3030, 2150 males and 880 females) [22], Demko et al. (3182) [23], Do et al. (3551) [24], Pentenero et al. (4098) [25], Avcu et al. (5150) [26], and Bhatnagar et al. (8866, 5187 males and 3679 females) [27].

The prevalence of OMLs was 21.08% in the study population which was comparable to the study done by Kumar et al. (18.89%) [14] and Do et al. (20.5%) [24], less than the studies done by Demko et al. (26.7%) [23], Castellanos and Díaz-Guzmán (35.66%) [28], Mumcu et al. (41.7%) [13], Vallejo et al. (51.1%) [29], Ali et al. (58.1%) [11], El Toum et al. (61.8%) [6], Blanco DC P et al. (76.9%) [12], and Chiang et al. (92.83%) [20], and higher than the studies by Saraswathi et al. (4.1%) [19], Ikeda et al. (4.9%) [30], Oivio et al. (8.58%) [5], Mehrotra et al. (8.4%) [22], Zain et al. (9.7%) [31], Nair et al. (14%) [32], Al-Mobeeriek et al. (15%) [21], Bhatnagar et al. (16.8%) [27], Parlak et al. (26.2%) [15], Jahanbani et al. (28%) [33], Ambika (64.11%) [34], and Yañez et al. (37.62%) [35], which were conducted among a very young group of population (children ranging from 0-16 years).

The most common OMLs seen among the study population were smoker's melanosis (7.79%), which was similarly seen in studies by Mumcu et al. (6.9%) [13] and Saraswathi et al. (1.14%) [19]; traumatic ulcer in the study by Castellanos and Díaz-Guzmán (4.02%) [28]; and smoker's palate in the study by Bhatnagar et al. (10.44%) [27]. 34.07% of tobacco habit-related lesions were seen in our study population; 4.1% of habit-related soft tissue lesions were seen Saraswathi et al. [19].

Tongue lesions were observed among 26.48% of our study population, and it was quite higher in studies by Avcu et al. (52.23%) [26], children from 1 to 14 years by Vörös-Balog et al. (35.11%) [36], Mumcu et al. (36.05%) [13], Koay et al. (30.2%) [37], Al-Mobeeriek et al. (3.96%) [21], and Carrard et al. (1.21%) [18].

The coated tongue in our study was seen in 4.17%, which was higher than the studies by Al-Mobeeriek et al. (0.55%) [21] and Kumar et al. (1.5%) [14] but relatively lower than the studies by Toum et al. (17.4%) [6] and Avcu et al. (23.24%) [26].

The prevalence of fissured tongue in our study was 3.43%, which was relatively low as compared to the study by Bhatnagar et al. (35.3%) [27] but higher than the studies by Kumar et al. (0.2%) [14] and Oivio et al. (1.1%) [5].

The malignancy seen in our study was relatively low, i.e., 0.14%, which was comparable with the study by Do et al. (0.15%) [24], Kumar et al. (0.2%) [14], and Mehrotra et al. (0.06%) [22], and it was higher in the studies by Amaral et al. (2.5%) [4], Blanco DC P et al. (2.8%) [12], Gambhir et al. (7.5%) [10], and Bajracharya et al. (13.5%) [8]. The significant percentage in the study by Gambhir et al. [10] and Bajracharya et al. [8] could be because the study was done among the histopathological samples received for biopsy.

The prevalence of oral leukoplakia was found to be 3.37% in our study population, which was similarly seen in studies by Kumar et al. (3.1%) [14] and Jahanbani (3.7%) [16], higher than studies by Oivio et al. (0.5%) [5], Saraswathi et al. (0.59%) [19], Carrard et al. (1.01%) [18], Pentenero et al. (1.15) [25], Ferreira et al. (2.3%) [38], Bhatnagar et al. (2.83%) [27], and Mehrotra et al. (2.9%) [22], but lower than the studies by El Toum et al. (5.1%) [6] and Chung et al. (7.44%) [39].

The prevalence of oral lichen planus was found to be 2.55% in our study population, which was higher than the studies by Saraswathi et al. (0.15%) [19], Mehrotra et al. (0.19%) [22], Al-Mobeeriek et al. (0.35%) [21], Jahanbani (0.5%) [16], Mumcu et al. (0.5%) [13], Bhatnagar et al. (0.8%) [27], Carrard et al. (1.02%) [18], Kumar et al. (1.3%) [14], Pentenero et al. (1.46) [25], Oivio et al. (1.5%) [5], and Ikeda et al. (1.8%) [30], and lower than the studies by Chung et al. (2.98%) [39] and Vallejo et al. (3.2%) [29] but was in a comparable range. It was higher than the global estimate of 1.01% [40].

The prevalence of oral submucous fibrosis was found to be 1.81% which was similar to the study by Bhatnagar et al. (1.97%) [27] and Kumar et al. (2.5%) [14] but higher than the studies done by Mehrotra et al. (0.69%) [22], Chung et al. (1.58%) [39], and Saraswathi et al. (0.55%) [19].

The overall prevalence of OPMDs was 7.76%, which was relatively low as compared to studies by Kumar et al. (5.63%) [14], Gambhir et al. (22.2%) [10], and Ferreira et al. (29.6%) [38].

Salivary gland disorders were found to be 0.34% in our study group, which was relatively low than the study by Gambhir et al. (2.9%) [10] and Amaral et al. (8.6%) [4].

Mucocutaneous lesions were present among the 0.15% of the study population, which is again relatively low as compared to studies by Gambhir et al. (1.8%) [10].

The prevalence of RAS was 6.32% which was relatively higher than studies by Oivio et al. (0.1%) [5], Al-Mobeeriek et al. (0.39%) [21], Zain et al. (0.5%) [31], Castellanos and Díaz-Guzmán (0.85%) [28], Mumcu et al. (1.2%) [13], Bhatnagar et al. (1.53%) [27], Pentenero et al. (1.73) [25], Vallejo et al. (1.9%) [29], Kumar et al. (2.0%) [14], and Chiang et al. (4.3%) [20].

The prevalence of oral candidiasis was 2.11% in our study population, which was comparable with the studies done by Kumar et al. (1.5%) [14], Bhatnagar et al. (1.61%) [27], and Castellanos and Díaz-Guzmán (2.01%) [28]. The prevalence of normal mucosal variants was 13.96% which was more than the studies by Al-Mobeeriek et al. (8.66%) [21].

The prevalence of Fordyce's granules was 8.84% in our study population, which was more than the studies by Oivio et al. (1.2%) [5] and El Toum et al. (3.9%) [6] but relatively less as compared to studies by Bhatnagar et al. (19.89%) [27], Ali et al. (20.4%) [11], and Chiang et al. (82.8%) [20].

The prevalence of leukoedema was 0.72% in our study population which was comparable to studies by Mumcu et al. (refer for more) (0.4%) [13] but relatively lower than the studies by Al-Mobeeriek et al. (3.37%) [21], Castellanos and Díaz-Guzmán (10.56%) [28] and Bhatnagar et al. (15.72%) [27].

### 4.1. Gender

The prevalence of OMLs among males was significantly high among males (63.11%) than females (36.88%) in our study population; similarly, in studies by Saraswathi et al. (63.80% males and 36.19% females) [19], Pentenero et al. (27.3% males and 22.89% females) [25], Avcu et al. (62.0% in males and 44.2% in females), and [26] Bhatnagar et al. (12.6% in males and 4.28% in females) [27], and in contrast, OMLs were most commonly found among the females in studies by Al-Mobeeriek et al. (57.7% females and 42.3% males) [21], and Castellanos and Díaz-Guzmán (1.4 : 1 male to female ratio) [28]. In contrast, studies done by El Toum et al. [6] and Mumcu G et al. [13] did not find any gender difference.

### 4.2. Age Groups

Children in the young age group, i.e., 0-20 years, had a very low prevalence of OMLs in our study population, i.e., 37.36%, as compared to the study by Nair et al. (64.4%) 32 but higher than the studies by Jahanbani et al. (28%) [33].

OMLs were most frequently seen among the age group of 41-50 years followed by 31-40 years, which was similarly seen in the study by Bajracharya et al. (31-40 years) [8].

Oral candidiasis was seen most commonly among the elderly population aged >61 years.

OPMDs, malignancies, and tobacco-associated lesions were found most commonly among 31-40 age groups, in contrast to the melanin pigmentation and fissured tongue seen in the study by Mumcu et al. [13] among the elderly population.

The prevalence of aphthous ulcer among 11-20 years was 0.79%, which was relatively low as compared to the study by Parlak et al. (3.6%) in the age group of 13-16 years only [15] and Yanez et al. (6.9%) in the age group of 3-13 years [35].

### 4.3. Location

The most common location for OMLs in our study was buccal mucosa (22.23%), which was similarly seen in the study by Bajracharya et al. (20.7%) [8] and higher than the studies by Gambhir RS (16.8%) [10], whereas lower lip was found by Blanco DC P et al. (20.8%) [12] and palate by Bhatnagar et al. (16.8%) [27].

The least common location for OMLs was the floor of the mouth (1.33%) in our study, which was different from gingiva in studies by Bajracharya et al. [8] and Bhatnagar et al. (0.4%) [27].

Among the normal variants, frictional keratosis (1617, 36.71%) and linea alba were seen most frequently (1502, 34.10%), whereas Fordyce's granules were most commonly seen in the study by Ali et al. (20.4%) [11].

The prevalence of OMLs was relatively higher among the studies that were done on records of biopsy [7, 8, 10], and they will be definitely as compared to the screening samples of regular dental patients as in our study. Compared to our study, variations and differences in the prevalence of various OMLs and normal variants have been noted. This could be attributed to the very different geographical location, and none of the studies have been done near our study sample, which is relatively higher than previous studies in different geographical locations. Few of the studies took only young children or middle-aged or elderly populations, and none took a wide range of age groups (all age populations). The differences in the mucosal lesion can also be attributed to different food habits, cultural practices, and living environments.

Our study's limitation was that the population was from a single tertiary centre in Nepal, which gives region-specific data. We would suggest a multicenter countrywide further study to have a more specific prevalence. Another limitation of this study was that it was done retrospectively, and the histopathological confirmation of all the lesions was not done.

## 5. Conclusion

It was concluded that there is a wide variation in the prevalence of oral mucosal lesions worldwide, and the prevalence is specific to a particular region only. Our study has concluded that the Nepalese population has a wide range of oral mucosal lesions and normal variants, and this study has attempted to have baseline data for the same. OMLs were seen most commonly among males and the 41-50 age group. The most common OML group is related to tobacco, among which smoker's melanosis is the most common one. Smoker's melanosis was the most predominant lesion among the males in the 50-60 year age group.

## Figures and Tables

**(a):** 

OMLs		*n*	Overall prevalence (%)	Prevalence among OMLs (%)
OPMDs	Homogeneous leukoplakia	325	3.11	5.38
	Nonhomogeneous leukoplakia	28	0.26	0.46
	Erythroplakia	3	0.02	0.04
	OSMF	189	1.81	3.13
	Lichen planus	267	2.55	4.42
	Discoid lupus erythematosus	1	0.006	0.01
	Lichenoid reaction	2	0.012	0.03

Malignancy	Squamous cell carcinoma	9	0.08	0.14

Tobacco-related	Smoker palate	631	6.04	10.45
	Smoker melanosis	814	7.79	13.49
	Tobacco pouch keratosis	544	5.21	9.01
	Betel chewer's mucosa	67	0.58	1.11

Ulcer	RAS	660	6.32	10.93
	Traumatic ulcer	68	0.65	1.12

Typical gingival	Gingival enlargement	107	1.02	1.77
	Pyogenic granuloma	1	0.006	0.01

Infectious	Candidiasis	221	2.11	3.66
	Herpes	29	0.27	0.48
	Herpes zoster	6	0.05	0.09

Mucocutaneous	Erythema multiforme	1	0.006	0.01
	Pemphigus	16	0.15	0.26

Salivary gland disorders	Pleomorphic adenoma	3	0.02	0.04
	Ranula	11	0.1	0.18
	Siadenitis	9	0.08	0.14
	Mucocele	15	0.14	0.24
	Necrotizing silaometaplasia	1	0.006	0.01

Tongue	Glossitis	94	0.9	1.55
	Geographic tongue	420	4.02	6.96
	Ankyloglossia	6	0.05	0.09
	Tongue depapillation	324	3.1	5.37
	Hairy tongue	64	0.61	1.06
	Fissured tongue	207	1.98	3.43
	Coated tongue	436	4.17	7.22
	Median rhomboid glossitis	47	0.45	0.77

Bone	Ameloblastoma	3	0.02	0.04
	Radicular cyst	33	0.31	0.54
	Residual cyst	5	0.04	0.08
	Dentigerous cyst	1	0.006	0.01
	OKC	2	0.012	0.03
	CEOT	1	0.006	0.01
	Mandibular tori	16	0.15	0.26
	Palatal torus	7	0.06	0.11

Soft tissue	Mucositis/stomatitis	109	1.04	1.8
	Capillary hemangioma	4	0.03	0.06
	Fibroma	45	0.43	0.74
	Lipoma	4	0.03	0.06
	Squamous papilloma	3	0.02	0.04
	Allergic angioedema	2	0.012	0.03

Others	Myositis	164	1.57	2.71
	Pulp polyp	8	0.07	0.13

**(b):** 

Normal variants	*n*	Overall prevalence (%)
Leukoedema	76	0.72
Fordyces granules	923	8.84
Melanosis	286	2.74
Frictional keratosis	1617	15.49
Linea alba	1502	14.39

**(a):** 

		Gender		
		Male	Female	Total
OPMD	Homogeneous leukoplakia	211	114	325
	Nonhomogeneous leukoplakia	21	7	28
	Erythroplakia	3	0	3
	OSMF	170	19	189
	Lichen planus	179	88	267
	Discoid lupus erythematosus	0	1	1
	Lichenoid reaction	1	1	2
	Total	585	230	815

Malignancy	SCC	7	2	9

Tobacco-associated lesions	Smoker palate	473	158	631
	Smoker melanosis	697	117	814
	Tobacco pouch keratosis	451	93	544
	Betel chewer's mucosa	58	9	67
	Total	1679	377	2056

Ulcer	RAS	287	373	660
	Traumatic ulcer	47	21	68
	Total	334	394	728

Typical gingival	Gingival enlargement	38	69	107
	Pyogenic granuloma	0	1	1
	Total	38	70	108

Infectious	Candidiasis	178	43	221
	Herpes	16	13	29
	Herpes zoster	4	2	6
	Total	198	58	256

Mucocutaneous	Erythema multiforme	0	1	1
	Pemphigus	4	12	16
	Total	4	13	17

Salivary gland lesions	Pleomorphic adenoma	1	2	3
	Ranula	7	4	11
	Sialadenitis	3	6	9
	Mucocele	7	8	15
	Necrotizing silaometaplasia	1	0	1
	Total	19	20	39

Tongue lesions	Glossitis	45	49	94
	Geographic tongue	236	184	420
	Ankyloglossia	2	4	6
	Tongue depapillation	109	215	324
	Hairy tongue	16	48	64
	Fissured tongue	116	91	207
	Coated Tongue	247	189	436
	Median rhomboid glossitis	26	21	47
	Total	797	801	1598

Bony lesions	Ameloblastoma	2	1	3
	Radicular cyst	14	19	33
	Residual cyst	3	2	5
	Dentigerous cyst	0	1	1
	OKC	1	1	2
	CEOT	0	1	1
	Mandibular tori	9	7	16
	Palatal torus	4	3	7
	Total	33	35	68

Soft tissue lesions	Mucositis/stomatitis	26	83	109
	Capillary hemangioma	2	2	4
	Fibroma	12	33	45
	Lipoma	3	1	4
	Squamous papilloma	2	1	3
	Allergic angioedema	0	2	2
	Total	45	122	167

Others	Myositis	67	97	164
	Pulp polyp	2	6	8
	Total	69	103	172

	Grand total	3808	2225	6033

**(b):** 

Normal variants	Male	Female	Total
Leukoedema	54	22	76
Fordyce's granules	596	327	923
Melanosis	89	197	286
Frictional keratosis	872	745	1617
Linea alba	859	643	1502
Total	2470	1934	4404

**(a):** 

Oral mucosal lesions	Age in years (10 years interval)							Total
	0 to 10	11 to 20	21 to 30	31 to 40	41 to 50	51 to 60	61 and >	
*OPMD*	*0*	*40*	*100*	*205*	*204*	*191*	*75*	
Homogeneous leukoplakia			24	86	92	81	42	325
Nonhomogeneous leukoplakia					11	14	3	28
Erythroplakia				2	1			3
OSMF		13	25	44	57	44	6	189
Lichen planus	0	27	51	71	43	51	24	267
Discoid lupus Erythematosus						1		1
Lichenoid reaction				2				2
*Malignancy*								0
SCC				1	2	2	4	9
*Tobacco-associated lesions*	*0*	*41*	*140*	*513*	*573*	*544*	*245*	0
Smoker palate		18	47	159	187	171	49	631
Smoker melanosis		17	68	213	232	221	63	814
Tobacco pouch keratosis		6	25	123	129	135	126	544
Betel chewer's mucosa				18	25	17	7	67
*Ulcer*	*22*	*85*	*260*	*83*	*105*	*102*	*71*	0
RAS	21	83	247	72	97	86	54	660
Traumatic ulcer	1	2	13	11	8	16	17	68
*Typical gingival*	*0*	*1*	*0*	*56*	*38*	*13*	*0*	*108*
Gingival enlargement				56	38	13		107
Pyogenic granuloma		1						1
*Infectious*	*15*	*14*	*29*	*31*	*45*	*33*	*89*	*256*
Candidiasis	14	8	19	23	41	29	87	221
Herpes	1	6	10	6	2	3	1	29
Herpes zoster				2	2	1	1	6
*Mucocutaneous*	*0*	*0*	*0*	*3*	*1*	*7*	*6*	*17*
Erythema multiforme							1	1
Pemphigus				3	1	7	5	16
*Salivary gland lesions*	*3*	*7*	*5*	*9*	*8*	*5*	*3*	*39*
Pleomorphic adenoma						1	2	3
Ranula		1	2	4	3	1		11
Sialadenitis		1	1	1	3	2	1	9
Mucocele	3	5	2	3	2	1		15
Necrotizing silaometaplasia				1				1
*Tongue lesions*	*17*	*50*	*228*	*370*	*481*	*305*	*100*	*1598*
Glossitis		1	2	14	19	31	27	94
Geographic tongue	15	27	87	99	111	46	35	420
Ankyloglossia	2		1	1	2			6
Tongue depapillation			17	112	138	52	5	324
Hairy tongue		1	6	12	19	22	4	64
Fissured tongue		14	33	65	79	13	3	207
Coated tongue		7	82	67	113	141	26	436
Median rhomboid glossitis								47
*Bony lesions*	*0*	*1*	*8*	*21*	*20*	*27*	*15*	*68*
Ameloblastoma			1	1	1			3
Radicular cyst			3	11	5	7		33
Residual cyst						3	2	5
Dentigerous cyst			1					1
OKC			1	1				2
CEOT					1			1
Mandibular tori		1	2	3	1			16
Palatal torus				5	12	17	13	7
*Soft tissue lesions*	*1*	*2*	*5*	*32*	*48*	*35*	*44*	*167*
Mucositis/stomatitis				21	32	19	37	109
Capillary hemangioma			0	1	1	1	1	4
Fibroma		2	5	8	12	13	5	45
Lipoma				1		2	1	4
Squamous papilloma					3			3
Allergic angioedema	1			1				2
*Others*	*0*	*28*	*44*	*49*	*19*	*22*	*10*	*172*
Myositis	0	21	43	49	19	22	10	164
Pulp polyp		7	1					8
Total	58 (0.96%)	269 (4.45%	819 (13.57%)	1373 (22.75%)	1544 (25.59%)	1286 (21.31%)	662 (10.97%)	6033

**(b):** 

Normal variants	Age in years (10 years interval)							Total
	0-10	11 to 20	21 to 30	31 to 40	41 to 50	51 to 60	61 and >	
Leukoedema			7	34	23	9	3	76
Fordyce's granules		27	87	211	314	257	27	923
Melanosis		16	73	95	46	41	15	286
Frictional keratosis		19	265	363	324	413	233	1617
Linea alba		23	257	282	419	367	154	1502
Total	0	85	689	985	1126	1087	432	4404

## Data Availability

The data used to support the findings of this study are available from the corresponding author upon request.
